# Some General Effects of War Upon the Voluntary Hospitals

**Published:** 1915-10-16

**Authors:** 


### SOME GENERAL EFFECTS OF WAR.*

### I. UPON THE VOLUNTARY HOSPITALS.

To what extent has the war affected (*a*) the finances; (*b*) the administration and other departments of typical voluntary hospitals?

This question is a very difficult one to answer. Even the hospital manager, if asked, would not find it easy to reply as regards his own institution except in very general terms. It is almost impossible to place one's finger upon the pulse of the voluntary system except at certain times and in certain conditions. Accurately to survey, for example, the finance of 1915 until the year 1916 is well on its way, and the reports of the hospitals are capable of tabulation, is not within the power of any statistician.

For a general survey of the situation, then, the only way open is to study the reports of the institutions for 1914 and to draw upon the impressions one has formed in constant intercourse with the officers of important hospitals. To draw upon the latter sources of information first, it can be said that the general effect of present conditions is (temporarily, of course) evidenced by either prosperity and great activity, or financial embarrassment and a comparatively eventless and monotonous course of affairs. Naturally, the exponent of the first state is an optimist and the other man a pessimist; but each has the good sense to see that the whole thing is a phase, and that a return to normal conditions is but a matter of time, in which day the pessimist will cheer up in the advent of the return of comfortable days.

### Increased Cost of Commodities.

In these times a deserving charity steadily pursuing its excellent work, where that work is not closely identified with stirring national events, is apt to be overlooked by the giving public. In other words, all the disadvantages of the situation are felt—the enormous increase in the prices of much-used medical and surgical stores, the smaller but substantial rise in the cost of foodstuffs, and the difficulty in obtaining suitable hospital employees to fill the places of those who are serving with the Forces or have been tempted away by some of the frequent opportunities of more remunerative employment. On the other hand, none of the advantage of the present-day civilian-military hospital, in seizing occasion for securing support from those not likely to give in peace times, is available, and the authorities are hard put to it to retain the favours of regular sympathisers. In such cases there is nothing for it but to hold tight and, recognising that the position is obviously unnatural, to wait for the recognition which should be given, sooner or later, to honest and necessary work for the good of the community.

To sum up the present position, one must rely largely upon observation and conjecture, but there are certain facts which in themselves illuminate. For some of these one may draw upon the reports of typical hospitals for 1914, containing statistics taken up to the end of the year and covering five months of conditions affected by the war, and the published opinions of executives when reporting to their governing bodies during the spring of the present year. These facts we propose to present in some detail in a second article.

Other concrete knowledge we possess in the records of prices paid for hospital stores at different periods, and in the experience of responsible people of the difficulty and delay in obtaining supplies of very necessary articles even at the prices quoted. The information we have gathered in respect of the markets as regards provisions and medical and surgical stores is for convenience arranged as follows:

*Prices which Should be Paid by a Hospital of Size.*

| | August 1, 1914 | | December 31, 1914 | | August 31, 1915 | |
|---|---|---|---|---|---|---|
| | s. | d. | s. | d. | s. | d. |
| **PROVISIONS.** | | | | | | |
| Meat—Beef (sirloin) per lb.... | 0 | 5½ | 0 | 7 | 0 | 9½ |
| Meat—Mutton (legs) per lb.... | 0 | 5½ | 0 | 7 | 0 | 8 |
| Bread, per 14 lbs. | 1 | 3 | 1 | 7¼ | 2 | 2¼ |
| Flour, per 14 lbs | 1 | 4½ | 1 | 9¼ | 2 | 8¼ |
| Butter (fresh), per lb. | 1 | 2 | 1 | 3 | 1 | 3 |
| Cheese, per lb. | 0 | 7½ | 0 | 9 | 0 | 9½ |
| Bacon, per lb. | 0 | 10 | 0 | 10½ | 0 | 10 |
| Eggs (new laid), per doz. | 1 | 3 | 2 | 6 | 1 | 8 |
| Tea, per lb. | 1 | 0½ | 1 | 7½ | 1 | 8¼ |
| Sugar (loaf), per cwt. | 16 | 0 | 36 | 0 | 34 | 0 |
| **MEDICAL AND SURGICAL STORES.** | | | | | | |
| Bismuth Carbonate, per lb.... | 8 | 9½ | 11 | 5 | 11 | 5 |
| Aspirin, per lb. | 1 | 9½ | 7 | 0 | 43 | 0 |
| Olive Oil, per gall. | 4 | 6 | 5 | 3 | 6 | 0 |
| Cotton Wool, per lb. | 0 | 5¾ | 0 | 6¾ | 0 | 8 |
| Chloroform, per lb. | 1 | 6 | 1 | 10 | 1 | 11 |
| Liquid paraffin, per gall. | 3 | 4 | 17 | 6 | 16 | 6 |
| Potass. bromide, per lb. | 1 | 6½ | 3 | 0 | 9 | 0 |
| Potass. iodide, per lb. | 11 | 5 | 12 | 5 | 14 | 3 |
| Cream of tartar, per lb. | 0 | 11¾ | 1 | 6 | 2 | 0 |
| Atropine sulphate, per oz. ... | 23 | 0 | — | | 95 | 0 |
| **BEDS.** | | | | | | |
| Each | 31 | 0 | — | | 34 | 0 |

It will be noticed that the figures in the third column relate to pre-Budget prices.

Another serious increase in expenditure is in the matter of salaries and wages. Remuneration of the resident medical staff, when an institution is so fortunate as to retain a full staff in this department, is often a question of individual bargaining; speaking generally, it is safe to say that the rates are quite double the scale of normal times. Dispensers are difficult to obtain, and their rates of pay are distinctly higher. The salaries on the nursing side are as a rule about the same, but the shortage of nurses often compels a hospital to engage temporary help at a cost which, for the purpose, is extravagant. Domestic servants, male and female, are not easy to get, and, when engaged, are often unsettled and hard to retain; the scale of pay,

* N.B.—Evidence of the effects of war upon other sections of the field of charity is invited.—Ed. "The Hospital.

although higher than formerly, is not greatly increased, but constant changes involve extra expense. The administrative officers, clerks, etc., being generally non-resident, feel the effects of the decreased value of money, and with increased work, owing to the changes brought about by the times, have not, so far as we have been able to learn, benefited by the general higher scale of remuneration applied to several other departments. Altogether it is obvious that the cost per occupied bed for 1915 will in most hospitals prove to be very high, and will only be modified where there has been a temporary important increase in the number of beds which will absorb part of the standing charges not included in the departments of expenditure we have surveyed.

